# Associations between loneliness and perceived social support and outcomes of mental health problems: a systematic review

**DOI:** 10.1186/s12888-018-1736-5

**Published:** 2018-05-29

**Authors:** Jingyi Wang, Farhana Mann, Brynmor Lloyd-Evans, Ruimin Ma, Sonia Johnson

**Affiliations:** 1 Division of Psychiatry – University College London, 6th Floor, Maple House, 149 Tottenham Court Road, London, W1T 7NF England; 2 Camden and Islington NHS Foundation Trust, St Pancras Hospital, 4 St Pancras Way, London, NW1 0PE England

**Keywords:** Loneliness, Perceived social support, Outcomes, Mental health problems, Systematic review

## Abstract

**Background:**

The adverse effects of loneliness and of poor perceived social support on physical health and mortality are established, but no systematic synthesis is available of their relationship with the outcomes of mental health problems over time. In this systematic review, we aim to examine the evidence on whether loneliness and closely related concepts predict poor outcomes among adults with mental health problems.

**Methods:**

We searched six databases and reference lists for longitudinal quantitative studies that examined the relationship between baseline measures of loneliness and poor perceived social support and outcomes at follow up. Thirty-four eligible papers were retrieved. Due to heterogeneity among included studies in clinical populations, predictor measures and outcomes, a narrative synthesis was conducted.

**Results:**

We found substantial evidence from prospective studies that people with depression who perceive their social support as poorer have worse outcomes in terms of symptoms, recovery and social functioning. Loneliness has been investigated much less than perceived social support, but there is some evidence that greater loneliness predicts poorer depression outcome. There is also some preliminary evidence of associations between perceived social support and outcomes in schizophrenia, bipolar disorder and anxiety disorders.

**Conclusions:**

Loneliness and quality of social support in depression are potential targets for development and testing of interventions, while for other conditions further evidence is needed regarding relationships with outcomes.

**Electronic supplementary material:**

The online version of this article (10.1186/s12888-018-1736-5) contains supplementary material, which is available to authorized users.

## Background

There is increasing interest in the effects of social relations on health, and in the service delivery and policy implications of such effects [1]. Loneliness has been a particularly prominent focus in recent research on physical health [2–4]. For instance, two meta-analytic reviews have reported that loneliness and poor social support are associated with higher mortality rates, and that the effect is comparable with some well-established risk factors such as obesity, physical inactivity, and smoking [5, 6]. They are also predictive of development of coronary heart disease and stroke [7], increases in systolic blood pressure [8, 9], and chronic pain [10, 11] in longitudinal studies. The effect of loneliness on physical health may be via biological, psychological and/or behavioural mechanisms, including physiological functioning, neuroendocrine effects, gene effects, immune functioning, perception of stressful events, health behaviours and sleep quality [2, 12, 13]. In contrast, while loneliness and lack of social support are well-documented problems among mental health service users [14], they have not been prominent in research, mental health service delivery and policy. Until recently, there has tended to be less focus on the social determinants of mental health than on genetics and neurobiology, but recent integrated aetiological models, such as the integrated sociodevelopmental-cognitive model, bring social factors into the neuroscientific mainstream, with increasing evidence that such factors need to be included to achieve models of good explanatory value [15–17]. An increasing focus on loneliness has also been driven by recognition of its high prevalence, and of its wide ranging impacts on physical health and mental well-being [17]. Despite this increasing recognition of its importance, to date no systematic synthesis of the evidence on the relationship between loneliness and the outcomes of mental health problems has been published.

Loneliness has been defined as a negative emotional state that occurs when there is “a discrepancy between…the desired and achieved patterns of social interaction” [18]. Loneliness is sometimes seen as an essentially unidimensional concept, sometimes as comprising two dimensions. Weiss [19] proposed a multidimensional concept of loneliness, categorising loneliness into social and emotional dimensions. Social loneliness derived from “the absence of socially integrative relationships”, while emotional loneliness stemmed from the absence of “a close emotional attachment” [19]. Social loneliness occurs when a person does not have a wider social network as desired, which can lead to the feelings of boredom, exclusion and marginality [19, 20]. In contrast, emotional loneliness occurs when someone is missing an intimate relationship, which can result in distress and apprehension [19, 20]. Psychometrically robust self-report measures of loneliness have been developed and used extensively in research on physical health and on older people, including the University of California at Los Angeles (UCLA) Loneliness Scale [21] and the de Jong-Gierveld Loneliness Scale [22]. Feelings of loneliness are more prevalent among people with mental illness than in the general population [23, 24]. In a study of older adults with major depression, dysthymia, or minor depression, 83% of the respondents reported loneliness and 38% reported severe loneliness [25]. By comparison, only 32% of non-depressed elderly people were lonely and 4% severely lonely using the same loneliness scale [26]. In a comparison of people with psychosis and a general population sample with similar demographic characteristics, the prevalence of loneliness among people with psychosis was 79.9% compared with 35% in the general population [27]. For people with depression, cross-sectional studies have found up to 40% of respondents feeling lonely most of the time [28], with a tenfold increase in the odds of being lonely compared to the general population [29].

Given the high prevalence of loneliness among people with mental health problems and the evidence for its harmful effects in other populations, good quality evidence is needed on its impact on recovery from mental health problems and on the health and social functioning of mental health service users. This has potential to inform the development of preventive and therapeutic interventions for which there is not as yet an evidence base. An important question in evaluating the available evidence is how far loneliness is conceptually and empirically distinct from other concepts and measures related to social relationships. Loneliness has been shown to be only moderately correlated with more objectively measured concepts such as social isolation, social network size and objective social support received from others [30, 31]. However, subjectively rated concepts related to social relationships are less easy to distinguish clearly from loneliness [32]. For example, perceived social support refers to people’s beliefs about how much support is potentially available from their relationships and social contacts and about the quality of this support [33, 34]. This is distinct from received social support, a rating of how often someone reports receiving particular supportive behaviours [33, 34]. Measures of perceived social support assess the quality or adequacy of social support from a subjective perspective. For instance, the two widely used measures, the Multi-dimensional Scale of Perceived Social Support (MSPSS) [35] and the Subjective Support Subscale of Duke Social Support Index (DSSI) [36], consist of items such as “How often do you feel lonely?”, “Can you talk about your deepest problems?”, “I have friends with whom I can share joys and sorrows”, which have a high degree of overlap with loneliness measures. Likewise, measures of confiding relationships assess the extent to which people feel close to and able to talk intimately with other people [37, 38]. Studies have found large negative correlations between loneliness and perceived social support [39–42]. Thus these concepts resemble loneliness as subjective evaluations of the quality and impact of social relationships: given this conceptual overlap, this paper includes them along with loneliness.

Three previous systematic reviews have explored the relationship between social relations and depression in general population [43, 44], or older adults [45], but included both cross-sectional and prospective studies. One further review looked at the relationship between social networks and support and early psychosis in people with first episode psychosis and in general population samples, but included no prospective studies [46]. To our knowledge, there is no systematic review which summarises and synthesises the evidence regarding the relationship between loneliness and perceived social support and the course of existing mental health problems, and which includes only prospective studies, from which inferences about the direction of causation may be drawn. Our review will fill this gap, and will provide useful evidence about how far and in what context loneliness and perceived social support may influence mental health recovery. Thus the aim of the current paper is to synthesise the available evidence as to whether higher levels of loneliness and poorer perceived social support have an adverse effect on outcomes in adults of all ages with existing mental health problems.

## Methods

A systematic review was conducted of the scientific literature addressing the question of whether loneliness and low perceived social support are associated longitudinally with poorer outcomes among adults of all ages with a range of mental health problems. The review’s protocol was registered on PROSPERO, which is an international database of prospective systematic reviews with health related outcomes (registration number: CRD42015014784) [47].

### Inclusion criteria

Types of study: The review included longitudinal studies in which the relationship between baseline measures of loneliness and poor perceived social support and outcomes at follow up was examined using quantitative measures.

Participants: Participants in the included studies were adults with mental illnesses, specifically schizophrenia and schizoaffective disorder, psychosis in general, depression, bipolar disorder, and anxiety disorders. Clinical populations were included however diagnosis was made, for example clinical diagnoses, ratings according to the criteria of the Diagnostic and Statistical Manual of Mental Disorders (DSM) or the International Classification of Diseases (ICD), or use of reliable and valid instruments such as the Mini-International Neuropsychiatric Interview (M.I.N.I.). We excluded studies with samples of children under 16 years old, people with intellectual disabilities or organic mental disorders including dementia, or cohorts assembled on the basis of a primary physical illness diagnosis.

Exposure variables: Included studies used quantitative measures of loneliness or of related concepts that involve a subjective rather than objective appraisal of social relationships, such as perceived social support or confiding relationships. Concepts based on objective ratings of the size and functioning of social networks, such as social isolation and social network size, were excluded. Social capital was also excluded as it relates to characteristics of society or communities as a whole as well as individuals’ appraisal of their relationships, and is conceptually distinct from loneliness [32]. We included studies only if exposure variables assessed subjective appraisal of overall social connectedness, rather than the quality of specific relationships: therefore, measures of support from partner and quality of a specific significant relationship were excluded.

Outcomes: The review included a wide variety of outcomes, ranging from clinical outcomes to functioning outcomes. Studies in which any of the following outcomes were measured at follow-up were eligible for inclusion:

1. Relapse: recurrent episodes following recovery at baseline of mental illness meeting the criteria of DSM or ICD, or of other reliable and valid instruments such as the Center for Epidemiologic Studies Depression Scale (CES-D), and proxy measures of acute relapse such as admission to psychiatric hospital/crisis services/acute mental health services.
2. Measures of functioning or of recovery: recovery of function, social functioning, self-rated recovery, quality of life, and disability.
3. Symptom severity: level of symptoms, symptom improvement or deterioration.
4. Global outcome: overall outcome rating combining different aspects of mental health and functioning, such as the Health of the Nation Outcome Scales (HoNOS).

### Search strategy

A systematic search of the following six electronic databases was undertaken: Medline, PsycINFO, Embase, Web of Science, CINAHL and Cochrane Library (1891 to April 2016). No language and publication period restrictions were applied. Search terms for loneliness and related concepts were combined with terms for mental disorders and outcomes. Searches were conducted using both subject headings (MeSH terms) and text words within title and abstract. Search terms were adapted as required for different databases (for full details, see Additional file 1). The search terms used in Medline are as follows:

1. Loneliness: loneliness [MeSH] OR loneliness OR lonely OR social support adj5 (subjective or personal or perceived or quality) OR confiding relationship*
2. Mental disorders: mental disorders [MeSH]. exp. OR mental OR psychiatr* OR schizo* OR psychosis OR psychotic OR depress* OR mania* OR manic OR bipolar adj5 (disorder or disease or illness) OR anxiety disorders [MeSH]. exp.
3. Outcomes: prognosis [MeSH] OR outcome* OR recurren* OR relapse OR admission OR hospitali?ation OR crisis OR admitted OR detained OR detention OR recovery of function [MeSH] OR “social functioning” OR “self-rated recovery” OR “quality of life” OR “symptom severity” OR disability

Apart from outcome terms, we searched “onset” and related terms as searches were conducted simultaneously for this and a companion systematic review on loneliness as a risk factor for the onset of psychiatric disorders in the general population. Reference lists of studies identified through the electronic search for inclusion in the review and of review articles were manually searched for further relevant studies. Relevant studies reported in dissertations, conference reports or other sources other than published journals were searched using the free text and keyword searches from the following two sources: Zetoc (indexing and abstracting database of conference proceedings) and OpenGrey (system for information on grey literature in Europe). When necessary and possible, we sent emails to authors to request full text or clarify some uncertainties.

Selection of studies for inclusion in the review was made independently by two reviewers (J.W. & F.M.). Titles of all identified studies were screened. The abstracts of potentially relevant studies were read; the full text of studies still considered potentially relevant was then retrieved and read. All studies included by one assessor were confirmed by the other reviewer to check adherence to inclusion criteria in study selection. 800 studies excluded by one assessor were checked by the other reviewer to establish reliability of our study selection. The agreement between reviewers was higher than 99%. Queries about inclusion/exclusion were resolved through discussion with a third reviewer (S.J.).

### Data extraction, quality assessment and synthesis

A structured template was developed to extract relevant data from eligible papers. Two review authors (J.W. & F.M.) independently extracted data and assessed their methodological quality. Extracted data and quality assessment scores were checked by a second reviewer for 20% of papers. Disagreements between the two assessors were resolved through discussion with a third review author (S.J.). The methodological quality of each study included in the review was assessed using a standard form adapted from the Mixed Methods Appraisal Tool (MMAT) – Version 2011 [48]. The MMAT has been designed for appraisal of the methodological quality for qualitative, quantitative and mixed methods studies. For quantitative studies it includes criteria relevant to randomised controlled, non-randomised, and descriptive studies. For the purposes of our review, we used the criteria for the quantitative non-randomised domain (Cohort study version). As there are four criteria for this domain following two screening questions, the overall quality score was presented using descriptors *, **, ***, and ****, ranging from * (one criterion met) to **** (all criteria met). The four criteria related to selection bias, measurement quality, adjustment for confounders, and percentage of complete outcome data/response rate/follow-up rate (see Additional file 2). We conducted a narrative synthesis of results as the anticipated heterogeneity of included studies, for example in samples, predictor measures and outcomes, made a meta-analysis inappropriate. The main results have been stratified by type of mental health problem investigated and tables and text were used to summarise the data.

## Results

### Literature search

**Fig. 1:**
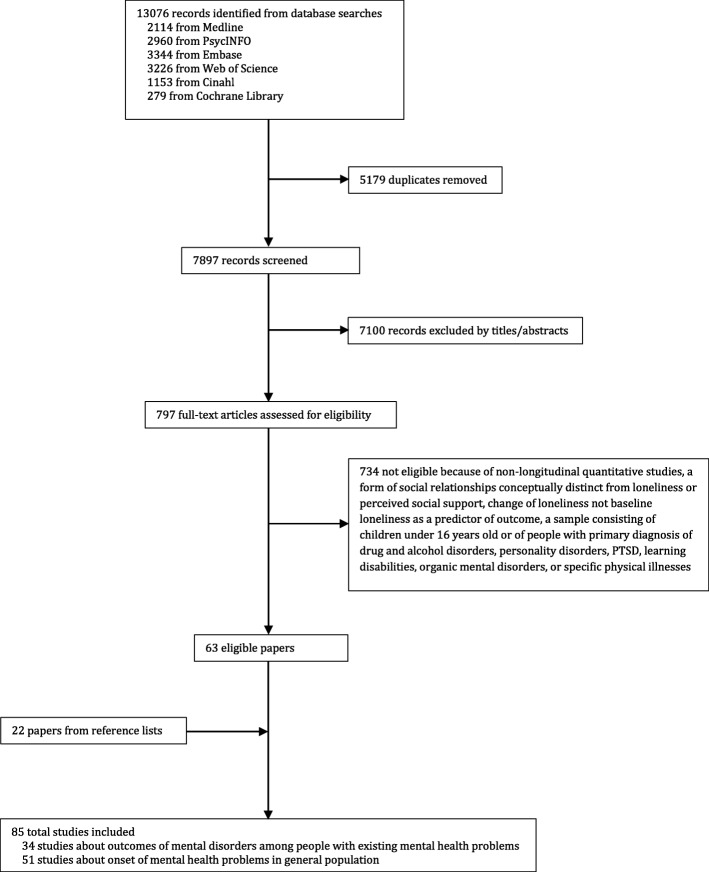
Studies selection flowchart

Our initial database search retrieved 13,076 records (see Fig. 1). After excluding duplicates and screening titles and abstracts to exclude obviously irrelevant papers, 797 full-text articles were assessed for eligibility. 734 studies were excluded because: i) they were not longitudinal quantitative studies; ii) they assessed a form of social relationships conceptually distinct from loneliness or perceived social support; iii) they analysed the relationship between change scores in loneliness and outcome variables, rather than baseline loneliness as a predictor of outcome; or iv) they investigated a sample consisting of children under 16 years old or of people with primary diagnoses of drug and alcohol disorders, personality disorders, post-traumatic stress disorder (PTSD), learning disabilities or organic mental disorders, or of people recruited as having specific physical illnesses. Twenty-two further papers were retrieved by hand-searching the reference lists of the papers already identified. Of the resulting 85 studies, 34 articles about outcomes of mental disorders among people with existing mental health problems were included in this review. The other 51 papers will be reported in a companion systematic review regarding the relationship between loneliness and onset of mental health problems in the general population. The search results are reported as a Prisma diagram in the Fig. 1.

### Eligible papers

The 34 eligible papers were from seven countries, including 23 from North America, 10 from Europe and one from Israel. These papers consisted of 23 studies with samples of people with depression, two focusing on schizophrenia or schizoaffective disorders, four on bipolar disorder, and three on anxiety disorders. Two further studies included people with a mixture of mental health problems (Table 1). Only two studies directly assessed loneliness, and most of the studies used various scales to measure perceived social support. Nearly half of included papers studied symptom severity as an outcome, a third of the papers assessed recovery/remission, and a third of the papers included other outcomes such as quality of life, disability pension qualification, functional impairment or life satisfaction. The sample sizes of six studies in our review exceeded 400, 22 were between 100 and 400, and six were less than 100. Six studies had short length of follow-up (less than one year), 23 following up the cohorts for one to two years, and five for over two years. With regard to quality assessment, five studies were assigned a maximum score of four (****) as their overall quality scores, 16 studies had a score of three (***) and 13 papers had two (**) according to the appraisal criteria of MMAT. Most studies had lower quality assessment ratings because they did not report the percentage of complete outcome data, response rate or follow-up rate (for full details, see Additional file 2).

**Table 1:** Summary of characteristics of included studies

Condition studied	Predictor variable	Outcomes	Sample size range (median)	Length of follow-up^a^	Follow-up rate range (median)	Quality score
Depression (*n* = 23)	Perceived social support (*n* = 22) Loneliness (*n* = 1)	Symptom severity (*n* = 13) Recovery/remission (*n* = 7) Functional outcomes (*n* = 5)	66–604 (239)	Short (*n* = 4) Medium (*n* = 14) Long (*n* = 5)	60.6–100% (81.9%)	**** (*n* = 4) *** (*n* = 11) ** (*n* = 8)
Schizophrenia/schizoaffective disorders (*n* = 2)	Perceived social support (*n* = 2)	Functional outcomes (*n* = 2)	139–148 (143.5)	Medium (*n* = 2)	71.9–100% (86.0%)	*** (*n* = 1) ** (*n* = 1)
Bipolar disorder (*n* = 4)	Perceived social support (*n* = 4)	Symptom severity (*n* = 3) Recovery/remission (*n* = 2) Functional outcomes (*n* = 2)	42–173 (55.5)	Short (*n* = 1) Medium (n = 3)	71.1–100% (86.4%)	*** (*n* = 2) ** (*n* = 2)
Anxiety disorders (*n* = 3)	Perceived social support (*n* = 3)	Symptom severity (*n* = 1) Recovery/remission (*n* = 1) Functional outcomes (*n* = 1)	134–1004 (1004)	Short (*n* = 1) Medium (*n* = 2)	80–87% (81.0%)	**** (*n* = 1) *** (*n* = 1) ** (*n* = 1)
Mixed samples with various mental health problems (n = 2)	Perceived social support (*n* = 1) Loneliness (*n* = 1)	Symptom severity (*n* = 1) Functional outcomes (*n* = 1)	352–743 (547.5)	Medium (*n* = 2)	79.9–84.4% (82.2%)	*** (*n* = 1) ** (*n* = 1)

^a^Length of follow-up: Short = < 1 year; Medium = 1–2 years; Long = > 2 years. ** = two criteria of MMAT met. *** = three criteria of MMAT met. **** = all criteria of MMAT met

### Depression

Among the 23 papers with samples of people with depression, 13 studies assessed depression severity as an outcome. Eleven of these found that poorer perceived social support or greater loneliness at baseline was a significant predictor of higher depressive symptom severity at follow-ups (Table 2). Nine of these eleven papers conducted multivariable analyses including adjusting for baseline depression severity. In eight of these nine papers, the relationship between baseline loneliness and depressive symptom outcome remained significant. For example, among the three studies with high quality scores (****), Blazer and colleagues [49] and Brugha and colleagues [50] followed cohorts of adults with depression in America and the UK respectively. They reported that poorer subjective social support at baseline was predictive of poorer outcomes at follow-up, outcomes including poorer life satisfaction (beta = 0.10, B = 0.37), worse depressive symptoms (beta = 0.10, B = 0.30) [49], and more severe psychiatric status (regression coefficient = − 1.46) [50]. In the third study rated as high quality, Leskela and colleagues [51] assessed adults with major depressive disorder and found that lower perceived social support six months after initial assessment predicted more severe depression at 18 months among all participants, although this relationship only remained significant in multivariable analysis for the group who had remitted following initial assessment (*r* = − 0.321). The only study using loneliness as a predictor of depression outcomes was conducted by Holvast and colleagues [25] among Dutch older adults. They found that a 1-point higher loneliness score was predictive of a 0.61-point higher depressive symptom severity score at follow-up (Beta = 0.61, 95% CI 0.12–1.11). For studies which reported beta as the effect size, beta ranged from 0.10 to 0.61. Among the 13 studies, three articles had high quality (****), four had medium quality (***), and the other six received low quality ratings (**). However, no obvious relationship was found between study quality and whether results were significant.

**Table 2:** Summary of findings on depression

Reference	Predictor variable	Outcome variable	Results (++ < 0·05 adjusted; + < 0·05 unadjusted; − non-significant)	
Hybels et al. (2016) [79]	Perceived social support	Trajectory class (quick recovery, slow recovery, persistent moderate, and persistent high)	++	Patients in the persistent moderate depression class had lower levels of baseline subjective social support compared with patients in the quick recovery class (OR (95%CI) = 0.91 (0.83, 0.98)). Patients in the persistent high depression class had lower levels of baseline subjective social support compared with those in the quick recovery class (OR (95%CI) = 0.83 (0.75, 0.92))
Holvast et al. (2015) [25]	Loneliness	Symptom severity; Remission	++ ++	In the fully adjusted model, a 1-point higher baseline loneliness score predicted a 0·61-point higher depressive symptom severity score at follow-up (Beta = 0.61, 95% CI 0.12–1.11, *p* = 0.02). Logistic regression analysis showed that while adjusting for social network size and potential confounders, the very severely lonely respondents were less likely to achieve remission from their depressive disorder compared with the non-lonely respondents (OR = 0.25, 95% CI 0.08–0.80, *p* = 0.02).
Holma et al. (2012) [54]	Perceived social support	Disability pensions	+	Lower perceived social support at baseline predicted greater likelihood of being granted a disability pension over 5 year follow-up on univariate analysis (*p* = 0.031), but not significant in multivariate analyses where the outcome was the interval time to the date the pension was granted
Backs-Dermott et al. (2010) [80]	Perceived social support	Relapse versus stable remitted	++	Lower perceived social support from a significant other (standardized discriminant function coefficient 0.48) and lower perceived social support from friends (standardized coefficient 0.35) at baseline predicted greater likelihood of depressive relapse at one-year follow-up. The Discriminant Function Analysis was significant, Wilk’s Lambda = 0.69, x^2^ (5) = 16.35, *p* = 0.006
Bosworth et al. (2008) [81]	Perceived social support	Depression severity	++	Poorer subjective social support was a significant predictor of more severe depression at 12 months. Standardized beta = − 0.13, *p* = 0.05
Rytsala et al. (2007) [55]	Perceived social support	Work disability allowances	+	Lower perceived social support at 6 month was a significant predictor of greater likelihood of being granted disability allowances at 18 months (F = 6.3, *p* = 0.013), but not significant in multivariate analysis
Rytsala et al. (2006) [56]	Perceived social support	Functional disability; Social and work adjustment; Days spent ill in bed or not	++ ++ −	Lower perceived social support at baseline was a significant predictor of more severe functional disability at 6 months (B = 0.232, *β* = 0.210, *p* = 0.002, 95% CI 0.084 to 0.379), and poorer social and work adjustment at 6 months (B = − 0.008, *β* = − 0.222, *p* = 0.001, 95% CI -0.013 to − 0.003). Lower perceived social support at 6 months was one of the most significant factors predicting more severe functional disability at 18 months (B = 0.240, *β* = 0.215, *p* = 0.002, 95% CI 0.088 to 0.393), and poorer social and work adjustment at 18 months (B = − 0.011, *β* = − 0.303, *p*‹0.001, 95% CI -0.015 to − 0.006). But perceived social support did not predict any days spent ill in bed or not
Leskela et al. (2006) [51]	Perceived social support	Severity of depression	+	Lower perceived social support at 6 months predicted more severe depression at 18 months in original zero-order correlation (*r* = − 0.392, *p* < 0.001) and within-group standardised correlation (*r* = − 0.230, *p* = 0.001) among all patients, but not significant in multivariate analysis. In full remission group at 6 months (*n* = 67), lower perceived social support at 6 months predicted higher level of depressive symptoms at 18 months in multivariate analysis (*r* = − 0.321, *p* = 0.012)
Steffens et al. (2005) [82]	Perceived social support	Severity of depression	++	Lower subjective social support at baseline predicted more severe depression over time (estimate − 0.5641, *p* = 0.0002)
Ezquiaga et al. (2004) [83]	Perceived social support	Episode remission	–	Higher perceived social support at baseline did not predict remission at 12 months in univariate analysis (*p* = 0.33), and it was not included in multivariate analysis
Reference	Predictor variable	Outcome variable	Results (++ < 0·05 adjusted; + < 0·05 unadjusted; − non-significant)	
Gasto et al. (2003) [84]	Perceived social support	Severity of residual symptoms	++	Lower subjective social support at baseline predicted higher intensity of residual symptoms at 9 months in remitters (standardized β = 0.41, *p* < 0.001)
Bosworth et al. (2002) [53]	Perceived social support	Time-to-remission	++	Lower subjective social support at baseline (Hazard Ratio = 0.47, 95% CI: 0.31–0.71, *p* = 0.003) was a significant predictor of longer time to remission
Bosworth et al. (2002) [52]	Perceived social support	Remission	++	Lower baseline levels of subjective social support (OR = 1.21, 95% CI: 1.09–1.35, *p* < 0.001) predicted poorer recovery one year later
Triesch (2002) [85]	Perceived social support	Severity of depressive symptoms; Quality of life	− −	Lower perceived social support at baseline did not predict more severe depression (β = − 0.17) or poorer quality of life (β = − 0.12) at 3 months
Hays et al. (2001) [57]	Perceived social support	Activities of daily living	++	There was modest support for hypothesis that baseline subjective social support predicted functional declines at 1 year. There was partial support for hypothesis that the buffering effects of social support against functional decline would be strongest among the most severely depressed patients
Oxman and Hull (2001) [86]	Perceived social support	Depression severity	++	Greater perceived social support predicted subsequent decreases in depression among participants randomly assigned to placebo group (6-week depression − 0.18, *p* < 0.05; 11-week depression − 0.22, *p* < 0.05), but not significant among paroxetine group or Problem-Solving Treatment for Primary Care group
Brummett et al. (2000) [87]	Perceived social support	Depressive symptoms	–	Higher levels of received support at baseline significantly predicted decreases in depressive symptoms at both 6 months and 1 year, whereas subjective support did not significantly predict changes in depressive symptoms at either point in time
Sherbourne et al. (1995) [88]	Perceived social support	Number of depressive symptoms	++	Decreased number of depressive symptoms between baseline and 2-year follow-up was predicted by social support at baseline (standardised regression coefficients = 0.12, zero-order Pearson product-moment correlations = 0.16, *p* < 0.05). Among the subset of patients who had current depressive disorder at baseline, perceived social support was not significantly related to remission. Among patients without current depressive disorder at baseline (subthreshold depression), patients with higher level of perceived social support were less likely to experience a new depressive episode during 2-year period: odds ratio = 0.96 (CI:0.95, 0.98)
Blazer et al. (1992) [49]	Perceived social support	Decreased life satisfaction symptoms; Endogenous symptoms	++	Impaired subjective support at baseline was predictive of poorer outcome at 12-month follow-up in both models: decreased life satisfaction symptoms (b = 0.10, B = 0.37, *p* ≤ 0.001), endogenous symptoms (b = 0.10, B = 0.30, *p* ≤ 0.01)
Blazer et al. (1991) [89]	Perceived social support	Depressive symptoms	+	Intercorrelation between social support at baseline and depression score at 6 months: − 0.41, *p* < 0.001. Intercorrelation between social support at baseline and depression score at 12 months: − 0.34, *p* < 0.001
Brugha et al. (1990) [50]	Perceived social support	Symptom severity	++	After controlling for the two significant clinical predictors, a significant main effect was found in total sample for lower satisfaction with support at baseline on more severe psychiatric status at 4 months (regression coefficient = − 1.46, *p* < 0.05)
George et al. (1989) [90]	Perceived social support	Depressive symptoms	++	Impaired subjective social support at baseline is a significant predictor of higher numbers of CES-D symptoms at follow-up (b = 8.88, B = 0.20, *p* ≤ 0.05)
Krantz and Moos (1988) [91]	Perceived social support	Remitted, partially remitted, and nonremitted	+	Lower quality of relationships at baseline predicted poorer remission status after 1 year (χ^2^ = 10.21, *p* < 0.01)

Six out of seven articles which used recovery/remission of depression as their outcomes reported lower perceived social support or higher loneliness at baseline as a significant predictor of lower rates of recovery/remission at follow-up. Three of the seven studies adjusted for baseline depression severity, and all of them reported significant results. For example, in the study of Holvast et al. [25], the lonely respondents at baseline were reported to be less likely to achieve remission from their depressive disorder at follow-up compared with the non-lonely respondents (OR = 0.25, 95% CI 0.08–0.80). Similarly, poorer perceived social support at baseline was a significant predictor of poorer recovery one year later (OR = 1.21, 95% CI 1.09–1.35) [52], and of longer time-to-remission in a study of initially depressed elderly individuals (Hazard Ratio = 0.47, 95% CI 0.31–0.71) [53]. However, none of the seven studies had high quality scores (****), with five receiving medium (***), and two low scores (**).

With regard to functional outcomes (five articles), three studies have found that lower perceived social support at baseline was a significant predictor of greater likelihood of being granted disability pensions during the follow-up period (no effect size reported) [54, 55] and of more severe functional disability (beta 0.210 to 0.215, 95% CI 0.084–0.393) [56]. There is also evidence that greater perceived social support predicted better social and work adjustment (beta − 0.222 to − 0.303, 95% CI -0.013 to − 0.006) [56], and buffered functional declines in performance on activities of daily living (no effect size reported) [57]. However, after adjustment for potential confounders only two [56, 57] of the five studies had significant results.

### Schizophrenia/schizoaffective disorders

Two studies assessed patients with schizophrenia or schizoaffective disorders to identify psychosocial predictors of health-related quality of life and functional outcomes (Table 3). Ritsner and colleagues [58] followed a sample of inpatients with schizophrenia or schizoaffective disorders for 16 months and found that greater support from friends at baseline predicted better satisfaction with life quality after 16 months (accounted for 2.9% of quality of life index scores at follow up examination). In an American study, greater perceived social support was a strong predictor of better scores on a social functioning domain (no effect size reported), although it did not predict the global functioning score (a composite of vocational and social functioning, and independent living) [59].However, neither of these studies adjusted for the outcome variable baseline scores.

**Table 3:** Summary of findings on schizophrenia and schizoaffective disorders

Reference	Predictor variable	Outcome variable	Results (++ < 0·05 adjusted; + < 0·05 unadjusted; − non-significant)	
Ritsner et al. (2006) [58]	Perceived social support	Quality of life	++	Higher friend support at baseline predicted better satisfaction with life quality after 16 months (accounted for 2.9% of quality of life index scores at follow up examination)
Brekke et al. (2005) [59]	Perceived social support	Global functional outcome (work, social functioning, and independent living); Social functioning domain	− ++	Higher social support did not significantly predict better global functional outcome at 12 months (*p* < 0.10). But social support became a much stronger and statistically significant predictor of social functioning domain

### Bipolar disorder

We found four papers that studied adults with a diagnosis of bipolar disorder (Table 4). The evidence regarding depressive symptoms was consistent and showed that lower perceived social support predicted greater depression over time (beta − 0.14 to − 0.25, regression coefficient − 1.33) [60–62]. Lower perceived support was also found to be a significant predictor of greater impairment in functioning (beta − 0.14 to − 0.67) [60, 61], and longer time to recovery (no effect size reported) [62]. Among remitted patients with prior diagnosis of bipolar I disorder, greater perceived social support reduced risk of recurrence of any type (depressive or manic) at one year (OR = 0.92, 95% CI 0.85–0.99) [63]. With regard to severity of manic symptoms, however, the results were not so consistent. In one study lower perceived support significantly predicted more severe manic symptoms on follow-up assessment (beta = − 0.32) [61], but in other two studies it was not linked with subsequent manic symptomatology [60, 62]. Apart from the study of recurrence, the other three had adjustment for baseline score on the outcome measure.

**Table 4:** Summary of findings on bipolar disorder

Reference	Predictor variable	Outcome variable	Results (++ < 0·05 adjusted; + < 0·05 unadjusted; − non-significant)	
Koenders et al. (2015) [60]	Perceived social support	Depressive symptomatology; Depression related functional impairment; Manic symptomatology; Manic related functional impairment	++ ++	Lower perceived support predicted more depression related functional impairment during the subsequent 3 months (*β* (SE) = − 0.14 (0.03), *p* < 0.001), and with more depressive symptomatology at the subsequent time point (*β* (SE) = − 0.14 (0.04), *p* = 0.002). No significant associations between perceived social support and manic symptoms and impairment were observed
			− −	
Cohen et al. (2004) [63]	Perceived social support	Recurrence	++	After controlling for clinical variables, lower social support of any kind significantly predicted recurrence of any type at one year (β (SE) = − 0.09 (0.04), *p* = 0.03, OR = 0.92, 95% CI 0.85–0.99)
Daniels (2000) [61]	Perceived social support	Depressive symptomatology; Manic symptomatology; Functional impairment	++ ++ ++	Lower perceived support was a significant predictor of more severe depressive symptomatology after controlling for initial levels of depression (R^2^ = 0.67, F = 34.15, ΔR^2^ = 0.05, ΔF = 5.24, beta = − 0.25). Lower perceived support significantly predicted more severe manic symptomatology over three months (R^2^ = 0.18, F = 3.74, ΔR^2^ = 0.10, ΔF = 4.18, beta = − 0.32). Lower perceived social support significantly predicted impairment in functioning in the participants who completed their life charts for 90 consecutive days, after controlling for initial levels of functional impairment (R^2^ = 0.44, F = 5.48, ΔR^2^ = 0.41, ΔF = 10.22, beta = − 0.67).
Johnson et al. (1999) [62]	Perceived social support	Time to recovery; Severity of depressive symptoms; Severity of manic symptoms	++ ++ −	Lower social support was a significant predictor of longer time to recovery in Cox regression survival analyses (χ2 (1, *N* = 52) change = 5.89, one-tailed *p* < 0.01). In hierarchical multiple regression analyses, low social support predicted higher depression over time (regression coefficient = − 1.33, *p* < 0.01, R^2^ change = 0.07, F change = 11.70). Social support did not have significant impact on mania score at 6-month follow-up

### Anxiety disorders

The three studies of patients with anxiety disorders all reported significant associations between perceived social support at baseline and outcomes at follow-up (Table 5). Two studies included people with diagnoses of generalised anxiety disorder, panic disorder, social anxiety disorder or post-traumatic stress disorder. One study found that lower perceived social support was predictive of more severe anxiety (beta = − 0.15, CI [− 0.30, − 0.06], Ratios 8.85%) and depressive symptoms (beta = − 0.16, CI [− 0.28, − 0.08], Ratios 10.51%) at subsequent time points [33], and the other one found that greater perceived social support predicted a higher rate of remission at 6-month follow-up (OR = 1.38, 95% CI Wald 1.09–1.75) [64]. In a study of older adults with generalised anxiety disorder, Shrestha et al. [65] found that individuals with greater perceived social support at baseline reported greater average quality of life over time (beta = 0.41), albeit without adjustment for the outcome variable baseline score.

**Table 5:** Summary of findings on anxiety disorders

Reference	Predictor variable	Outcome variable	Results (++ < 0·05 adjusted; + < 0·05 unadjusted; − non-significant)	
Jakubovski and Bloch (2016) [64]	Perceived social support	Remission; Response (a reduction of at least 40% symptoms at 6 months)	++ ++	Generalised anxiety disorder: Greater amount of social support predicted a higher rate of remission (OR = 1.38, 95% CI Wald 1.09–1.75, *p* = 0.0067) and a greater rate of response (OR = 1.33, 95% CI Wald 1.10–1.62, *p* = 0.0040) at 6-month follow-up. Social anxiety disorder: Greater amount of social support predicted a higher rate of remission (OR = 1.716, 95% CI Wald 1.028–2.867, *p* = 0.0391) at 6-month follow-up, but social support did not predict response. Social support did not predict remission or response for panic disorder or post-traumatic stress disorder
Shrestha et al. (2015) [65]	Perceived social support	Quality of life	++	Main effect of social support was significant such that those with higher baseline social support reported higher average quality of life over time (b (SE) = 0.41 (0.08), *p* < 0.001)
Dour et al. (2014) [33]	Perceived social support	Anxiety symptoms; Depressive symptoms	++ ++	Direct effects: Relations between perceived social support and depression were bidirectional at all follow-ups, whereas they were unidirectional between perceived social support and anxiety at 6- and 12-month follow-ups. Indirect effects: Intervention led to changes in 6- and/or 12-month perceived social support, that in turn led to subsequent changes in 18-month depression (*b* = − 0.16, CI [− 0.28, − 0.08], Ratios 10.51%) and anxiety (*b* = − 0.15, CI [− 0.30, − 0.06], Ratios 8.85%)

### Mixed samples with various mental health problems

Two studies examined mixed samples of people with a variety of diagnoses (Table 6). Beljouw et al. [66] analysed data from primary care patients with current anxiety or depressive disorders, and found that greater loneliness at baseline was predictive of more severe depressive (beta = 0.89) or anxiety symptoms (beta = 0.40) at 1-year follow-up. However, after adjustment for baseline severity of depression or anxiety, only the relationship with depression severity remained significant (beta = 0.39). Fleury and colleagues [67] conducted a study among individuals with severe mental health problems including schizophrenia and other psychotic disorders and severe mood disorders. They reported that greater perceived social support was significantly predictive of higher subjective quality of life at 18 months (beta 0.136 to 0.196, 95% CI 0.255 to 3.410). However, adjustments for baseline measures included functional ability in the community and diagnosis, but not baseline quality of life.

**Table 6:** Summary of findings on mixed samples with various mental health problems

Reference	Predictor variable	Outcome variable	Results (++ < 0·05 adjusted; + < 0·05 unadjusted; − non-significant)	
Fleury et al. (2013) [67]	Perceived social support	Subjective quality of life	++	Social support variables at baseline accounted for 7.9% of quality of life at 18-month follow-up. Among social support dimensions, higher perception of availability of social integration (β = 0.196, *t* = 3.472, *p* = 0.001, 95% CI [0.942, 3.410]) and reassurance of worth supports (β = 0.136, *t* = 2.397, *p* = 0.017, 95% CI [0.255, 2.597]) at baseline predicted better quality of life at 18-month follow-up
Van Beljouw et al. (2010) [66]	Loneliness	Severity of depression; Severity of anxiety	++ +	A higher symptom severity in depression at 1-year follow-up was predicted by more loneliness at baseline in both multilevel univariate linear regression analyses (β = 0.89, SE = 0.17, *p* < 0.001) and multilevel multivariate linear regression analyses (β = 0.39, SE = 0.16, *p* < 0.05). Positive associations were found between more symptom severity in anxiety at 1-year follow-up and loneliness at baseline by multilevel univariate linear regression analyses (β = 0.40, SE = 0.12, *p* < 0.01) (but not significant in multivariate analyses)

## Discussion

### Main findings

We found 34 studies that reported quantitatively on the longitudinal relationship between perceived social support/loneliness at baseline and various outcomes of mental illness at follow-up. Although substantial heterogeneity exists in the identified articles, some generalisations can be made. There is substantial evidence that less perceived social support at baseline tends to predict greater symptom severity, poorer recovery/remission and worse functional outcomes at follow-up among people with depression, and preliminary evidence of a similar relationship for people with bipolar disorder, or anxiety disorders. There is also some evidence that greater loneliness is associated with more severe depression and anxiety symptoms and poorer remission from depression. An important consideration in interpreting findings is that depression is very likely to make people more likely to appraise their social support as inadequate and to feel emotionally lonely. However, a persistent effect on outcomes is found in many studies with adjustment for baseline depression severity. With regard to schizophrenia/schizoaffective disorders, only functional outcomes have been studied and the small amount of available evidence suggests that greater perceived social support is predictive of better subjective quality of life and social functioning. This review, to our knowledge, is the first to systematically examine longitudinal studies regarding the relationship between loneliness and closely related concepts and outcomes for adults of all ages and all types of mental illness.

### Strengths and limitations of the included studies and of this review

Generally, the quality of included studies is acceptable and most studies were assigned at least *** as their overall quality scores in accordance with the methodological quality criteria of MMAT. However, some methodological issues in the published literature may limit what can be inferred from the studies. Many studies did not have comprehensive information about percentage of complete outcome data, baseline response rate, or follow-up rate, resulting in lower quality assessment ratings. We did assess whether studies adjusted for baseline measurements on the outcomes. Some did not, increasing uncertainty about the direction of causation (although if the baseline outcome measure which included random errors was introduced as a covariate, regression to the mean might lead to biased results according to Lord’s paradox [68]). A large majority of the 23 studies that did adjust for baseline outcome measures still found loneliness/perceived social support to be predictive of outcomes. This suggests that there is a real effect of loneliness/lack of social support on outcomes. However, it is likely that the relationship can be a circular one, with loneliness/lack of social support resulting in more severe symptoms, and more severe symptoms exacerbating loneliness/lack of social support.

The consistency of findings across a variety of settings, measures of the exposure, and population groups increases confidence in the generalisability of the review’s findings. The retrieved articles encompassed varying populations including older and younger groups, and people recruited in primary care, inpatient and outpatient settings, and were carried out around the world. Most studies of perceived social support used well-developed scales where psychometric properties have been established. The measures used varied regarding the dimensions and types of social support assessed, although they all measure individuals’ subjective appraisal of adequacy or impact of their relationships rather than objective or structural social support. Both loneliness studies used a published measure of loneliness with well-established psychometric properties, but this review shows that knowledge about the relationship between loneliness and outcomes of mental health problems is still very limited. Finally, the studies in our review had sample sizes ranging from 42 to 1004, and diverse follow-up periods from a few months to ten years. The sample size of most studies is under 400 with less than 100 participants in six articles. However, the consistency of positive findings from included studies, irrespective of their sample size, provides some confidence that studies were not underpowered.

Other limitations of this review relate to the search strategy. Although our literature search was conducted in six databases and a variety of search terms were applied, the search might not be exhaustive. Some relevant studies may have been missed if they did not use “subjective or personal or perceived or quality” five or fewer words apart from “social support”. Some very old papers might not be indexed in electronic databases, and thus cannot be searched. Eligible studies are only from seven countries and most of them were conducted in North America. Very few papers in other languages were retrieved and none of them could be included in our review, although we did search for them and read their abstracts. It is also worth noting that the extent of any reporting bias is uncertain as studies which did not find a positive result might not be published. Another limitation refers to the scope of our review. We restricted the search to the most common mental disorders, including schizophrenia and schizoaffective disorder, psychosis in general, depression, bipolar disorder, and anxiety disorders. The associations between loneliness and perceived social support and other mental health problems need further investigation. Additionally, we focused on one direction of causation only: the effect of baseline loneliness and poor perceived social support on mental health outcomes at follow up. Psychiatric symptoms probably also influence loneliness and perceived social support, but this was not the research question on which we focused in this review.

### Research implications

Most studies included in our review focused on depression, with other types of mental health problems represented by fewer than five studies each. Nevertheless, some significant relationships have been found between loneliness and/or perceived social support and outcomes of those mental disorders. Gayer-Anderson and Morgan [46] systematically examined evidence on social networks and social support in early psychosis. They found some tentative evidence that deficits in social networks and support preceded the onset of psychosis, but it was difficult to disentangle direction of causation as almost all the studies included were cross-sectional and they did not report whether social relationships influence outcomes of psychosis. Given that the prevalence of loneliness in people with psychosis was comparable to that in people with depression, it is surprising that research about impact of loneliness/perceived social support on psychosis is scarce. Similarly, social relationships were shown to be related to bipolar disorder and anxiety disorders, but there is a lack of evidence to discern cause and effect [69, 70]. Therefore more systematic exploration is needed about how loneliness and perceived social support affect conditions such as psychosis, bipolar disorder and anxiety disorders.

Additionally, more longitudinal research with long-term follow-up (and repeated measures) is essential to untangle the direction of effect in the relationship between loneliness/perceived social support and poor outcomes. Among the 34 eligible studies only five articles involve a long-term follow-up period (over 2 years). Thus there is a need to establish the longer term associations of loneliness and perceived social support. As well its effects on longer term mental health outcomes, loneliness may contribute to the adverse physical health outcomes and increased mortality of people with severe mental health problems.

We also found that the relationship between perceived social support and depression was studied far more often and is thus far more clearly established than the relationship between loneliness and depression. Only two studies retrieved for our review included loneliness as an independent variable for outcomes of mental disorders. They found that loneliness at baseline predicted depression and anxiety severity and remission from depression [25, 66]. However, the few longitudinal studies of loneliness do not allow definitive conclusions. Therefore more longitudinal research is needed in clinical samples to try to achieve a clear understanding of the impact of loneliness on the course of mental health problems.

### Clinical and policy implications

There are a number of clinical and policy implications from the finding that poor perceived social support has a significant impact on outcomes in depression. Firstly, it highlights the need to pay sufficient attention to the social relationships and social support needs of people with mental health problems. Social activities, or thinking about relationships, can be overlooked in clinical consultations – in favour of medications or psychological therapies, and there have been recent calls to raise the profile of social factors in mental health care and mental health research [16]. Raising practitioners’ awareness of the beneficial effects of good perceived social support on symptoms, recovery, and functioning is an important first step, but also promoting awareness amongst service users and the wider public – so that people may feel more motivated to seek relevant help or to try to change their own situation, particularly in depression but probably in other mental health problems studied too.

The development of effective interventions to promote social support and reduce loneliness is required to address the current evidence gap, manifested by the absence of recommendations in this important social domain in current policy guidance. In the UK for example, the National Health Service (NHS) Five Year Forward View [71] refers to a series of plans to improve the quality of mental health services and reduce ‘burden’ on the NHS. Access to psychological therapies, waiting standards and better physical healthcare are highlighted but there is no specific mention of managing the significant problems of loneliness or limited social relationships. International evidence that poor perceived support from social relationships leads to increased service use and poorer outcomes across a range of diagnostic groups should inform future policy in this area. Also in the UK, the latest National Institute for Clinical Excellence (NICE) guidance on illnesses such as depression and schizophrenia, does not recommend social interventions apart from employment support [72, 73].

Clinicians may doubt whether loneliness and limited support from interpersonal relationships are appropriate or feasible as targets for intervention. However, potential interventions are becoming available in a variety of sectors. Around the world, approaches are being developed to try to reduce loneliness among older people in the general population, with potential to be adapted to other groups in the population at risk of adverse effects from poor social support. In the UK, a variety of approaches to social relationships and social participation are being developed primarily in the charitable sector and in primary care [74]. Social prescribing projects have proliferated in the UK in recent years [75]. Social prescribing is not precisely defined, but typically refers to: navigation - the process of linking support for people to access community activities helpful to wellbeing and participation; and/or funding and providing these activities in a community or group setting [76]. As yet however, social prescribing models are numerous and poorly defined [75], and there is a lack of robust evidence regarding their effectiveness [76]. Psychological approaches, such as Cognitive Behavioural Therapy and Mindfulness, have also been used to help people change their thinking about social relationships: some promising results have been reported, especially with older adult populations [77]. Thus there are approaches available with potential to be adapted and tested for people with mental health problems, to try to alleviate the adverse effects identified in this paper. There is also a need to consider public understanding of the importance of nurturing social relationships, as the high prevalence of loneliness is not only an individual but necessarily also a community and societal level problem. Thus people with mental health problems, like other groups in the population who are vulnerable to the effects of loneliness, are likely to benefit from an approach to loneliness that also takes account of community resources and how they might be enhanced [78].

## Conclusions

This systematic review has identified prospective studies in the area of loneliness/perceived social support and outcomes of mental health problems. We found substantial evidence that in depression, poorer perceived social support is associated with poorer outcomes in terms of symptoms, recovery and functioning. There is some preliminary evidence of a similar relationship in bipolar and anxiety disorders, and of a relationship between greater perceived social support and better quality of life and functioning in schizophrenia. Loneliness and its impact on mental health outcomes are still insufficiently addressed compared to perceived social support, but there is some evidence that greater loneliness is related to more severe depression and anxiety symptoms and poorer remission from depression. Further research, including long-term follow-up and repeated measures, is required to longitudinally investigate the direction of effect between loneliness/perceived social support and poor mental health outcomes among service users from different diagnostic groups. There is also a case, especially in depression, for raising public awareness of the importance of reducing public health, and for developing, testing and implementing strategies to improve perceived social support and reduce loneliness in people with mental health problems.

## Additional files

Additional file 1: Search strategies (detailed search strategies used in Medline, PsycINFO, Embase, Web of Science, CINAHL and Cochrane Library). (PDF 323 kb)

Additional file 2: Criteria for quality assessment and study evaluation table (study description table including author, year and country of each publication, study population, sample size, length of follow-up period, follow-up rate achieved, predictor variable, outcome variable and Study quality assessment rating). (PDF 280 kb)

## Abbreviations

CES-D: Center for Epidemiologic Studies Depression Scale
DSM: Diagnostic and Statistical Manual of Mental Disorder
HoNOS: Health of the Nation Outcome Scales
ICD: International Classification of Diseases
M.I.N.I.: Mini-International Neuropsychiatric Interview
MMAT: Mixed Methods Appraisal Tool
NHS: National Health Service
NICE: National Institute for Clinical Excellence
PTSD: post-traumatic stress disorder
UCLA: University of California at Los Angeles Loneliness Scale
