# Supplementary material for: Associations between loneliness and perceived social support and outcomes of mental health problems: a systematic review
Source: BMC Psychiatry. 2018 May 29;18:156. doi: 10.1186/s12888-018-1736-5 (PMC5975705; doi:10.1186/s12888-018-1736-5)
Supplement: Supplementary file 1 — Search strategies (detailed search strategies used in Medline, PsycINFO, Embase, Web of Science, CINAHL and Cochrane Library). (PDF 323 kb) [file 12888_2018_1736_MOESM1_ESM.pdf]

Additional file 1: Search strategies

Medline

Default search limits = title and abstract (except where otherwise stated)		
**#**	**Search term**	**Description**
1	loneliness [MeSH]	
2	loneliness	
3	lonely	
4	(social support adj5 (subjective or personal or perceived or quality))	
5	"confiding relationship*"	
6	#1 OR #2 OR #3 OR #4 OR #5	Loneliness and related terms
7	mental disorders [MeSH]. exp	
8	mental	
9	psychiatr*	
10	schizo*	
11	psychosis	
12	psychotic	
13	depress*	
14	mania*	
15	manic	
16	(bipolar adj5 (disorder or disease or illness))	
17	anxiety disorders [MeSH]. exp	
18	#7 OR #8 OR #9 OR #10 OR #11 OR #12 OR #13 OR #14 OR #15 OR #16 OR #17	Mental disorders
19	#6 AND #18	Loneliness, mental disorders
20	prognosis [MeSH]	
21	outcome*	
22	recurren*	
23	relapse	
24	admission	
25	hospitali?ation	
26	crisis	
27	admitted	
28	detained	
29	detention	
30	recovery of function [MeSH]	
31	"social functioning"	
32	"self-rated recovery"	
33	"quality of life"	
34	"symptom severity"	
35	disability	
36	#20 OR #21 OR #22 OR #23 OR #24 OR #25 OR #26 OR #27 OR #28 OR #29 OR #30 OR #31 OR #32 OR #33 OR #34 OR #35	Outcomes
37	onset	
38	first-episode	
39	incidence [MeSH]	
40	rate	
41	#37 OR #38 OR #39 OR #40	Onset
42	#36 OR #41	Outcomes/onset
43	#19 AND #42	Loneliness, mental disorders, outcomes/onset

PsycINFO

Default search limits = title and abstract (except where otherwise stated)		
**#**	**Search term**	**Description**
1	loneliness [Subject Headings]	
2	loneliness	
3	lonely	
4	(social support adj5 (subjective or personal or perceived or quality))	
5	“confiding relationship*”	
6	#1 OR #2 OR #3 OR #4 OR #5	Loneliness and related terms
7	mental disorders [Subject Headings]. exp	
8	mental	
9	psychiatr*	
10	schizo*	
11	psychosis	
12	psychotic	
13	depress*	
14	mania*	
15	manic	
16	(bipolar adj5 (disorder or disease or illness))	
17	anxiety disorders [Subject Headings]. exp	
18	#7 OR #8 OR #9 OR #10 OR #11 OR #12 OR #13 OR #14 OR #15 OR #16 OR #17	Mental disorders
19	#6 AND #18	Loneliness, mental disorders
20	prognosis [Subject Headings]	
21	outcome*	
22	recurren*	
23	relapse	
24	admission	
25	hospitali?ation	
26	crisis	
27	admitted	
28	detained	
29	detention	
30	recovery (Disorders) [Subject Headings]	
31	“social functioning”	
32	“self-rated recovery”	
33	“quality of life”	
34	“symptom severity”	
35	disability	
36	#20 OR #21 OR #22 OR #23 OR #24 OR #25 OR #26 OR #27 OR #28 OR #29 OR #30 OR #31 OR #32 OR #33 OR #34 OR #35	Outcomes
37	onset	
38	first-episode	
39	incidence	
40	rate	
41	#37 OR #38 OR #39 OR #40	Onset
42	#36 OR #41	Outcomes/onset
43	#19 AND #42	Loneliness, mental disorders, outcomes/onset

Embase

Default search limits = title and abstract (except where otherwise stated)		
**#**	**Search term**	**Description**
1	loneliness [Subject Headings]	
2	loneliness	
3	lonely	
4	(social support adj5 (subjective or personal or perceived or quality))	
5	“confiding relationship*”	
6	#1 OR #2 OR #3 OR #4 OR #5	Loneliness and related terms
7	mental disease [Subject Headings]. exp	
8	mental	
9	psychiatr*	
10	schizo*	
11	psychosis	
12	psychotic	
13	depress*	
14	mania*	
15	manic	
16	(bipolar adj5 (disorder or disease or illness))	
17	anxiety disorder [Subject Headings]. exp	
18	#7 OR #8 OR #9 OR #10 OR #11 OR #12 OR #13 OR #14 OR #15 OR #16 OR #17	Mental disorders
19	#6 AND #18	Loneliness, mental disorders
20	prognosis [Subject Headings]	
21	outcome*	
22	recurren*	
23	relapse	
24	admission	
25	hospitali?ation	
26	crisis	
27	admitted	
28	detained	
29	detention	
30	convalescence [Subject Headings]	
31	“social functioning”	
32	“self-rated recovery”	
33	“quality of life”	
34	“symptom severity”	
35	disability	
36	#20 OR #21 OR #22 OR #23 OR #24 OR #25 OR #26 OR #27 OR #28 OR #29 OR #30 OR #31 OR #32 OR #33 OR #34 OR #35	Outcomes
37	onset	
38	first-episode	
39	incidence [Subject Headings]	
40	rate	
41	#37 OR #38 OR #39 OR #40	Onset
42	#36 OR #41	Outcomes/onset
43	#19 AND #42	Loneliness, mental disorders, outcomes/onset

Web of Science

Default search limits = topic (except where otherwise stated)		
**#**	**Search term**	**Description**
1	loneliness	
2	lonely	
3	“social support” near/5 (subjective or personal or perceived or quality)	
4	“confiding relationship*”	
5	#1 OR #2 OR #3 OR #4	Loneliness and related terms
6	mental	
7	psychiatr*	
8	schizo*	
9	psychosis	
10	psychotic	
11	depress*	
12	mania*	
13	manic	
14	bipolar near/5 (disorder or disease or illness)	
15	anxiety	
16	#6 OR #7 OR #8 OR #9 OR #10 OR #11 OR #12 OR #13 OR #14 OR #15	Mental disorders
17	#5 AND #16	Loneliness, mental disorders
18	prognosis	
19	outcome*	
20	recurren*	
21	relapse	
22	admission	
23	hospitali?ation	
24	crisis	
25	admitted	
26	detained	
27	detention	
28	recovery	
29	“social functioning”	
30	“quality of life”	
31	“symptom severity”	
32	disability	
33	#18 OR #19 OR #20 OR #21 OR #22 OR #23 OR #24 OR #25 OR #26 OR #27 OR #28 OR #29 OR #30 OR #31 OR #32	Outcomes
34	onset	
35	first-episode	
36	incidence	
37	rate	
38	#34 OR #35 OR #36 OR #37	Onset
39	#33 OR #38	Outcomes/onset
40	#17 AND #39	Loneliness, mental disorders, outcomes/onset

Cinahl

Default search limits = title and abstract (except where otherwise stated)		
**#**	**Search term**	**Description**
1	loneliness [Subject Headings]	
2	loneliness	
3	lonely	
4	"social support" N5 (subjective or personal or perceived or quality)	
5	"confiding relationship*"	
6	#1 OR #2 OR #3 OR #4 OR #5	Loneliness and related terms
7	mental disorders [Subject Headings]. exp	
8	mental	
9	psychiatr*	
10	schizo*	
11	psychosis	
12	psychotic	
13	depress*	
14	mania*	
15	manic	
16	bipolar N5 (disorder or disease or illness)	
17	anxiety disorders [Subject Headings]. exp	
18	#7 OR #8 OR #9 OR #10 OR #11 OR #12 OR #13 OR #14 OR #15 OR #16 OR #17	Mental disorders
19	#6 AND #18	Loneliness, mental disorders
20	prognosis [Subject Headings]	
21	outcome*	
22	recurren*	
23	relapse	
24	admission	
25	hospitali?ation	
26	crisis	
27	admitted	
28	detained	
29	detention	
30	recovery [Subject Headings]	
31	"social functioning"	
32	"self-rated recovery"	
33	"quality of life"	
34	"symptom severity"	
35	disability	
36	#20 OR #21 OR #22 OR #23 OR #24 OR #25 OR #26 OR #27 OR #28 OR #29 OR #30 OR #31 OR #32 OR #33 OR #34 OR #35	Outcomes
37	onset	
38	first-episode	
39	incidence [Subject Headings]	
40	rate	
41	#37 OR #38 OR #39 OR #40	Onset
42	#36 OR #41	Outcomes/onset
43	#19 AND #42	Loneliness, mental disorders, outcomes/onset

Cochrane Library

Default search limits = title, abstract and keywords (except where otherwise stated)		
**#**	**Search term**	**Description**
1	loneliness [MeSH]	
2	loneliness	
3	lonely	
4	“social support” near/5 (subjective or personal or perceived or quality))	
5	“confiding relationship*”	
6	#1 OR #2 OR #3 OR #4 OR #5	Loneliness and related terms
7	mental disorders [MeSH]. exp	
8	mental	
9	psychiatr*	
10	schizo*	
11	psychosis	
12	psychotic	
13	depress*	
14	mania*	
15	manic	
16	bipolar near/5 (disorder or disease or illness)	
17	anxiety disorders [MeSH]. exp	
18	#7 OR #8 OR #9 OR #10 OR #11 OR #12 OR #13 OR #14 OR #15 OR #16 OR #17	Mental disorders
19	#6 AND #18	Loneliness, mental disorders
20	prognosis [MeSH]	
21	outcome*	
22	recurren*	
23	relapse	
24	admission	
25	hospitali?ation	
26	crisis	
27	admitted	
28	detained	
29	detention	
30	recovery of function [MeSH]	
31	“social functioning”	
32	“self-rated recovery”	
33	“quality of life”	
34	“symptom severity”	
35	disability	
36	#20 OR #21 OR #22 OR #23 OR #24 OR #25 OR #26 OR #27 OR #28 OR #29 OR #30 OR #31 OR #32 OR #33 OR #34 OR #35	Outcomes
37	onset	
38	first-episode	
39	incidence [MeSH]	
40	rate	
41	#37 OR #38 OR #39 OR #40	Onset
42	#36 OR #41	Outcomes/onset
43	#19 AND #42	Loneliness, mental disorders, outcomes/onset
