# Supplementary material for: Associations between loneliness and perceived social support and outcomes of mental health problems: a systematic review
Source: BMC Psychiatry. 2018 May 29;18:156. doi: 10.1186/s12888-018-1736-5 (PMC5975705; doi:10.1186/s12888-018-1736-5)
Supplement: Supplementary file 2 — Criteria for quality assessment and study evaluation table (study description table including author, year and country of each publication, study population, sample size, length of follow-up period, follow-up rate achieved, predictor variable, outcome variable and Study quality assessment rating). (PDF 280 kb) [file 12888_2018_1736_MOESM2_ESM.pdf]

Additional file 2: Criteria for quality assessment and study evaluation table

Criteria for quality assessment

The criteria for assessing study quality using the Mixed Methods Appraisal Tool (MMAT) [1] were clarified for this review as follows:

Screening questions

- Are there clear quantitative research questions (or objectives)?
- Do the collected data allow address the research question (objective)?

If the answer is not 'Yes' to one or both screening questions, further appraisal may be not feasible or appropriate and the paper would be excluded.

Methodological quality criteria

i) Are participants (organisations) recruited in a way that minimises selection bias?
At recruitment stage: Consider whether the exposed and non-exposed groups are recruited from the same population.

ii) Are measurements appropriate (clear origin, or validity known, or standard instrument) regarding the exposure and outcomes?
At data collection stage: Consider whether (a) the variables are clearly defined and accurately measured; (b) the measurements are justified and appropriate for answering the research question; and (c) the measurements reflect what they are supposed to measure.

iii) Are the most important factors which should be adjusted for taken into account in the analysis?
At data analysis stage: Consider whether (a) demographic characteristics are adjusted for, e.g. age, gender, marital status et al.; (b) outcomes at baseline are adjusted for, e.g. symptom severity at baseline.

iv) Are there complete outcome data (80% or above), and, when applicable, an acceptable response rate (60% or above), or an acceptable follow-up rate for cohort studies (60% or above)?

Studies included in review

Reference	Study population	Sample size	Length of follow-up period (short/medium/long term) (short = <1 year; medium = 1-2 years; long = >2 years)	Follow-up rate achieved	Predictor variable	Outcome variable	Study quality assessment rating [unmet criteria *]
**Depression**							
Hybels et al. (2016) [2], USA	Older adults with major depression	368	Long	Not clear	Perceived social support	Trajectory class (quick recovery, slow recovery, persistent moderate, and persistent high)	** [3, 4]
Holvast et al. (2015) [3], the Netherlands	Older adults with major depression, dysthymia, or minor depression	378	Medium	75.4%	Loneliness	Severity and remission of depression	*** [4]
Holma et al. (2012) [4], Finland	Psychiatric patients with major depressive disorder	269	Long	85.1% at 6 months, 77% at 18 months, 67.7% at 5 years	Perceived social support	Disability pensions	****
Backs-Dermott et al. (2010) [5], Canada	Female remitted depressed adults	90	Medium	64.4%	Perceived social support	Relapse versus stable remitted	*** [3]
Bosworth et al. (2008) [6], USA	Older adults with major depression	241	Medium	100%	Perceived social support	Depression severity	** [1, 4]
Rytsala et al. (2007) [7], Finland	Adults with diagnosis of unipolar depression	269	Medium	77.3%	Perceived social support	Work disability allowances	*** [4]
Rytsala et al. (2006) [8], Finland	Psychiatric patients with unipolar depression	269	Medium	87%	Perceived social support	Functional disability, social and work adjustment, and days spent ill in bed or not	*** [4]
Leskela et al. (2006) [9], Finland	Adults with major depressive disorder	269	Medium	85.1% at 6 months, 76.9% at 18 months	Perceived social support	Severity of depression	****
Steffens et al. (2005)	Older adults with major	204	Long	Not clear	Perceived social support	Severity of depression	**

[10], USA	depression						[1, 4]
Ezquiaga et al. (2004) [11], Spain	Adults with unipolar major depression	72	Medium	79.2%	Perceived social support	Episode remission	** [3, 4]
Gasto et al. (2003) [12], Spain	Elderly patients with unipolar major depression	108	Short	Not clear	Perceived social support	Severity of residual symptoms	** [3, 4]
Bosworth et al. (2002) [13], USA	Older adults with major depression	239	Long	86.5%	Perceived social support	Time-to-remission	*** [4]
Bosworth et al. (2002) [14], USA	Older adults with major depression	301	Medium	84.6%	Perceived social support	Remission	*** [4]
Triesch (2002) [15], USA	Adults with major depressive disorder	66	Short	68.0%	Perceived social support	Severity of depressive symptoms, and quality of life	*** [4]
Hays et al. (2001) [16], USA	Older adults with major depression	159	Medium	Not clear	Perceived social support	Activities of daily living	*** [4]
Oxman and Hull (2001) [17], USA	Older adults with dysthymia or minor depression	415	Short	74.0%	Perceived social support	Depression severity	*** [4]
Brummett et al. (2000) [18], USA	Older adults with major depression	115	Medium	94.8% at 6 months, 76.5% at 1 year	Perceived social support	Depressive symptoms	** [1, 4]
Sherbourne et al. (1995) [19], USA	Patients with depression/depressive symptoms	604	Medium	62%	Perceived social support	Number of depressive symptoms	** [2, 4]
Blazer et al. (1992) [20], USA	Adults with depression	118	Medium	98%	Perceived social support	Decreased life satisfaction symptoms, and endogenous symptoms	****
Blazer et al. (1991) [21], USA	Patients with depression	125	Medium	Not clear	Perceived social support	Depressive symptoms	** [3, 4]
Brugha et al. (1990) [22], UK	Adults with depression	130	Short	92%	Perceived social support	Symptom severity	****

George et al. (1989) [23], USA	Middle-aged and elderly depressed in-patients	150	Long	100%	Perceived social support	Depressive symptoms	*** [1]
Krantz and Moos (1988) [24], USA	Patients with major or minor depression	424	Medium	98.8%	Perceived social support	Remitted, partially remitted, and nonremitted	*** [3]
**Schizophrenia/schizoaffective disorders**							
Ritsner et al. (2006) [25], Israel	Inpatients with schizophrenia/schizoaffective disorders	148	Medium	100%	Perceived social support	Quality of life	*** [3]
Brekke et al. (2005) [26], USA	Adults with schizophrenia/schizoaffective disorders	139	Medium	71.9%	Perceived social support	Global functional outcome (work, social functioning, and independent living), and social functioning domain	** [3, 4]
**Bipolar disorder**							
Koenders et al. (2015) [27], the Netherland	Bipolar I and II outpatients	173	Medium	71.1%	Perceived social support	Symptoms severity, and functional impairment	*** [4]
Cohen et al. (2004) [28], USA	Remitted patients with prior diagnosis of bipolar I disorder	52	Medium	100%	Perceived social support	Recurrence	** [3, 4]
Daniels (2000) [29], USA	Adults with diagnosis of bipolar disorder	42	Short	95.2%	Perceived social support	Depressive symptomatology, manic symptomatology, and functional impairment	*** [1]
Johnson et al. (1999) [30], USA	Adults with diagnosis of bipolar disorder	59	Medium	77.6%	Perceived social support	Time to recovery, severity of manic and depressive symptoms	** [1, 4]
**Anxiety disorders**							
Jakubovski and Bloch (2016) [31], USA	Patients with diagnosis of generalized anxiety disorder, panic disorder, social anxiety disorder or post-traumatic stress	1004	Short	Not clear	Perceived social support	Remission, and response (a reduction of at least 40% symptoms at 6 months)	*** [4]

	disorder						
Shrestha et al. (2015) [32], USA	Older adults with generalised anxiety disorder	134	Medium	Not clear	Perceived social support	Quality of life	** [3, 4]
Dour et al. (2014) [33], USA	Adults with diagnoses of panic, generalized anxiety, social anxiety, and/or posttraumatic stress disorder	1004	Medium	87% at 6 months, 81% at 12 months, and 80% at 18 months	Perceived social support	Anxiety symptoms, and depressive symptoms	****
**Mixed samples with various mental health problems**							
Fleury et al. (2013) [34], Canada	Individuals with severe mental disorders according to the DSM-IV – schizophrenia and other psychotic disorders, or mood disorders	352	Medium	84.4%	Perceived social support	Subjective quality of life (satisfaction with life domains)	** [3, 4]
Van Beljouw et al. (2010) [35], the Netherlands	People with anxiety or depressive disorder	743	Medium	79.9%	Loneliness	Severity of depression and anxiety	*** [4]

* Quality criteria: 1 = selection bias; 2 = measurement quality; 3 = adjustment of confounders; 4 = percentage of complete outcome data/response rate/follow-up rate

Reference

1. Pluye P, Robert E, Cargo M, et al. Proposal: A Mixed Methods Appraisal Tool for Systematic Mixed Studies Reviews. 2011. http://mixedmethodsappraisaltoolpublic.pbworks.com/ (accessed Mar 27 2015).
2. Hybels CF, Pieper CF, Blazer DG, Steffens DC. Heterogeneity in the three-year course of major depression among older adults. *Int J Geriatr Psychiatr* 2016; **31**(7): 775-82.
3. Holvast F, Burger H, de Waal MMW, van Marwijk HWJ, Comijs HC, Verhaak PFM. Loneliness is associated with poor prognosis in late-life depression: Longitudinal analysis of the Netherlands study of depression in older persons. *J Affect Disord* 2015; **185**: 1-7.
4. Holma I, Holma K, Melartin T, Rytsala H, Isometsa E. A 5-year prospective study of predictors for disability pension among patients with major depressive disorder. *Acta Psychiatr Scand* 2012; **125**(4): 325-34.
5. Backs-Dermott BJ, Dobson KS, Jones SL. An evaluation of an integrated model of relapse in depression. *J Affect Disord* 2010; **124**(1-2): 60-7.
6. Bosworth HB, Voils CI, Potter GG, Steffens DC. The effects of antidepressant medication adherence as well as psychosocial and clinical factors on depression outcome among older adults. *Int J Geriatr Psychiatry* 2008; **23**(2): 129-34.
7. Rytsala H, Melartin T, Leskela U, Sokero T, Lestela-Mielonen P, Isometsa E. Predictors of long-term work disability in Major Depressive Disorder: A prospective study. *Acta Psychiatr Scand* 2007; **115**(3): 206-13.
8. Rytsala HJ, Melartin TK, Leskela US, Lestela-Mielonen PS, Sokero T, Isometsa ET. Determinants of Functional Disability and Social Adjustment in Major Depressive Disorder: A Prospective Study. *J Nerv Ment Dis* 2006; **194**(8): 570-6.
9. Leskela U, Rytsala H, Komulainen E, et al. The influence of adversity and perceived social support on the outcome of major depressive disorder in subjects with different levels of depressive symptoms. *Psychol Med* 2006; **36**(6): 779-88.
10. Steffens DC, Pieper CF, Bosworth HB, et al. Biological and social predictors of long-term geriatric depression outcome. *Int Psychogeriatr* 2005; **17**(1): 41-56.
11. Ezquiaga E, Garcia-Lopez A, de Dios C, Leiva A, Bravo M, Montejo J. Clinical and psychosocial factors associated with the outcome of unipolar major depression: a one year prospective study. *J Affect Disord* 2004; **79**(1-3): 63-70.
12. Gasto C, Navarro V, Catalan R, Portella MJ, Marcos T. Residual symptoms in elderly major depression remitters. *Acta Psychiatr Scand* 2003; **108**(1): 15-9.
13. Bosworth HB, McQuoid DR, George LK, Steffens DC. Time-to-remission from geriatric depression: Psychosocial and clinical factors. *Am J Geriatr Psychiatry* 2002; **10**(5): 551-9.
14. Bosworth HB, Hays JC, George LK, Steffens DC. Psychosocial and clinical predictors of unipolar depression outcome in older adults. *Int J Geriatr Psychiatry* 2002; **17**(3): 238-46.
15. Triesch SK. Interpersonal expectations and adjustment to depression. *Diss Abstr Int B Sci Eng* 2002; **63**(3-B): 1578.
16. Hays JC, Steffens DC, Flint EP, Bosworth HB, George LK. Does social support buffer functional decline in elderly patients with unipolar depression? *Am J Psychiatry* 2001; **158**(11): 1850-5.
17. Oxman TE, Hull JG. Social support and treatment response in older depressed primary care patients. *J Gerontol B Psychol Sci Soc Sci* 2001; **56**(1): P35-45.
18. Brummett BH, Barefoot JC, Siegler IC, Steffens DC. Relation of subjective and received social support to clinical and self-report assessments of depressive symptoms in an elderly population. *J Affect Disord* 2000;

61(1-2): 41-50.
19. Sherbourne CD, Hays RD, Wells KB. Personal and psychosocial risk factors for physical and mental health outcomes and course of depression among depressed patients. *J Consult Clin Psychol* 1995; **63**(3): 345-55.
20. Blazer D, Hughes DC, George LK. Age and impaired subjective support. Predictors of depressive symptoms at one-year follow-up. *J Nerv Ment Dis* 1992; **180**(3): 172-8.
21. Blazer D, Hughes DC. Subjective social support and depressive symptoms in major depression: separate phenomena or epiphenomena. *J Psychiatr Res* 1991; **25**(4): 191-203.
22. Brugha TS, Bebbington PE, Maccarthy B, Sturt E, Wykes T, Potter J. Gender, social support and recovery from depressive disorders: a prospective clinical study. *Psychol Med* 1990; **20**(1): 147-56.
23. George LK, Blazer DG, Hughes DC, Fowler N. Social support and the outcome of major depression. *Br J Psychiatry* 1989; **154**: 478-85.
24. Krantz SE, Moos RH. Risk factors at intake predict nonremission among depressed patients. *J Consult Clin Psychol* 1988; **56**(6): 863-9.
25. Ritsner M, Gibel A, Ratner Y. Determinants of changes in perceived quality of life in the course of schizophrenia. *Qual Life Res* 2006; **15**(3): 515-26.
26. Brekke J, Kay DD, Lee KS, Green MF. Biosocial pathways to functional outcome in schizophrenia. *Schizophr Res* 2005; **80**(2-3): 213-25.
27. Koenders MA, Giltay EJ, Hoencamp E, Elzinga BM, Spinhoven P, Spijker AT. The bidirectional impact of perceived and enacted support on mood in bipolar outpatients: A two-year prospective study. *Compr Psychiat* 2015; **60**: 59-67 9p.
28. Cohen AN, Hammen C, Henry RM, Daley SE. Effects of stress and social support on recurrence in bipolar disorder. *J Affect Disord* 2004; **82**(1): 143-7.
29. Daniels RE. Psychosocial predictors of symptom severity and functioning in adults with bipolar disorder: A three-month prospective study. *Diss Abstr Int B Sci Eng* 2000; **60**(8-B): 4213.
30. Johnson SL, Winett CA, Meyer B, Greenhouse WJ, Miller I. Social support and the course of bipolar disorder. *J Abnorm Psychol* 1999; **108**(4): 558-66.
31. Jakubovski E, Bloch MH. Anxiety Disorder-Specific Predictors of Treatment Outcome in the Coordinated Anxiety Learning and Management (CALM) Trial. *Psychiatr Q* 2016; **87**(3): 445-64.
32. Shrestha S, Stanley MA, Wilson NL, et al. Predictors of change in quality of life in older adults with generalized anxiety disorder. *Int Psychogeriatr* 2015; **27**(7): 1207-15.
33. Dour HJ, Wiley JF, Roy-Byrne P, et al. Perceived social support mediates anxiety and depressive symptom changes following primary care intervention. *Depress Anxiety* 2014; **31**(5): 436-42.
34. Fleury MJ, Grenier G, Bamvita JM, Tremblay J, Schmitz N, Caron J. Predictors of quality of life in a longitudinal study of users with severe mental disorders. *Health Qual Life Outcomes* 2013; **11**(1).
35. van Beljouw IM, Verhaak PF, Cuijpers P, van Marwijk HW, Penninx BW. The course of untreated anxiety and depression, and determinants of poor one-year outcome: a one-year cohort study. *Bmc Psychiatry* 2010; **10**: 86.
